# The association between various menstrual disorders and well-being was mediated by anxiety: a cross-sectional study

**DOI:** 10.1186/s12905-025-04053-y

**Published:** 2025-10-22

**Authors:** Chuan-Rong Yeh, Han T. Vo, Cheng-Yu Lin, Heng-Kien Au, Chih-Feng Lai, Sheng-Chih Chen, Tuyen V. Duong

**Affiliations:** 1 Master Program of Teaching Profession, National Taichung University of Education, Taichung, 40306 Taiwan; 2 International Ph.D. Program in Medicine, College of Medicine, Taipei Medical University, Taipei, 110-31 Taiwan; 3 Department of Psychiatry, Hue University of Medicine and Pharmacy, Hue University, Hue, 491-20 Vietnam; 4 Department of Radio, Television & Film, Shih Hsin University, Taipei, 116-42 Taiwan; 5 Department of Obstetrics and Gynecology, College of Medicine, Taipei Medical University, Taipei, 110-31 Taiwan; 6 Department of Education, National Taichung University of Education, Taichung, 403-06 Taiwan; 7 Graduate Program in Digital Content and Technologies, College of Communication, National Chengchi University, Taipei, 116-05 Taiwan; 8 School of Nutrition and Health Sciences, Taipei Medical University, Taipei, 110-31 Taiwan

**Keywords:** Menstrual disorders, Premenstrual symptoms, Intermenstrual bleeding, Abnormal uterine bleeding, Menstrual irregularities, Missed menstrual periods, Dysmenorrhea, Future anxiety, Well-being, Mediation analysis

## Abstract

**Abstract:**

Menstrual disorders are common health concerns that can negatively impact psychological well-being. This study examined the mediating role of future anxiety in the relationship between various menstrual disorders and well-being.

**Methods:**

A cross-sectional study was conducted among 399 Taiwanese women aged 18–48 from June 2021 to May 2022. Participants reported their menstrual cycles during the last six months. Individuals suffer from menstrual disorders if they have one of the following symptoms: infrequency, irregularity, abnormal flow volume, intermenstrual bleeding, pain/cramps, premenstrual symptoms, and one or more missed menstrual periods. Anxiety was evaluated using the Dark Future Scale. Well-being was measured using the World Health Organization’s Well-being Index.

**Results:**

Mediation analysis revealed that premenstrual symptoms were directly associated with well-being (coefficient (B) = -4.39, *p* = 0.018) and indirectly via future anxiety (B = -2.22, 95% confidence interval (CI) [-3.77, -0.86]). Additionally, the indirect effect of menstrual pains/cramps on well-being through future anxiety was significant, as the 95% CI did not include zero (B = -2.14, 95% CI [-3.81 to -0.71]). Intermenstrual bleeding between periods and abnormal light bleeding have indirect effects on well-being only via future anxiety (B = -1.84, 95% CI [-3.47, -0.41] and B = -1.58, 95% CI [-3.24, -0.06], respectively). However, for missed, infrequent, or irregular menstrual periods, future anxiety was not a significant mediator. We did not observe a significant relationship between heavy and well-being.

**Conclusions:**

This study highlights that future anxiety partially mediates the link between premenstrual symptoms and well-being, suggesting both direct and indirect effects. Intermenstrual bleeding, abnormal light bleeding, and menstrual pains/cramps have no direct impact on well-being but influence well-being only through future anxiety. However, missed, infrequent, or heavy/prolonged bleeding or irregular menstrual periods showed no significant association with well-being.

**Supplementary Information:**

The online version contains supplementary material available at 10.1186/s12905-025-04053-y.

## Abstract (plain language summary)

Menstrual disorders are common and can affect women’s well-being. This study examined how anxiety about the future may contribute to mediating the relationship between menstrual problems and overall well-being.

Researchers surveyed 399 Taiwanese women aged 18 to 48 between June 2021 and May 2022. The women answered questions about their menstrual cycles over the past six months. Participants also completed questionnaires measuring their level of future anxiety and general well-being.

The study found that women who experienced premenstrual symptoms reported lower well-being, both directly and because they felt more anxious about the future. Pain or cramps during menstruation were related to lower well-being, but this relationship happened only indirectly through future anxiety. Similarly, light bleeding between periods and unusual light bleeding were linked to lower well-being, but only because they increased future anxiety.

On the other hand, missed, infrequent, or irregular periods, as well as heavy bleeding, were not significantly linked to well-being, either directly or through anxiety.

In summary, the study suggests that anxiety about the future plays an important role in how certain menstrual symptoms affect a woman’s overall well-being. Helping women manage future-related anxiety might improve their mental health, especially for those dealing with pain, premenstrual symptoms, or irregular bleeding patterns.

**Supplementary Information:**

The online version contains supplementary material available at 10.1186/s12905-025-04053-y.

## Introduction

Menstrual disorders are an umbrella term to describe abnormal conditions related to menstrual cycles, such as dysmenorrhea (painful menstruation), abnormal menstrual bleeding, infrequent menstruation, irregular menstruation, and premenstrual syndrome [1]. As many as 87% of women in their childbearing age experience at least one menstrual disorder [2, 3]. Menstrual disorders (MDs) are a common health problem in women of reproductive age. These conditions impact physical health, leading to chronic discomfort, disruptions to daily life, and a decline in overall well-being. Irregular menstruation is associated with chronic diseases, such as diabetes, metabolic syndrome, coronary heart disease, or infertility [4]. Heavy menstrual bleeding was associated with shortness of breath, tiredness, and worse self-reported physical health [5].

There is evidence that MDs not only impact physical issues but also lead to mental health problems such as anxiety, depression, and stress, which can lower well-being. People with dysmenorrhea or heavy menstrual bleeding have more distress, irritability, and emotional exhaustion and are at high risk of anxiety and depression, reducing overall well-being [6, 7]. The underlying pathophysiology may involve fluctuation in estrogen and progesterone levels during the menstrual cycle, which can affect the balance of neurotransmitters such as serotonin, gamma-aminobutyric acid, and impair brain plasticity, contributing to a higher risk of depression and anxiety disorders [8]. Moreover, severe menstrual symptoms can interfere with education, work productivity, daily activities, and social interactions [9, 10]. Thereby, it compounds their psychological and functional burden.

One psychological factor that may help explain the mental health impact of MDs is future anxiety, which was defined as the feeling of excessive worry, tension, or fear about uncertain or potentially negative future events. A person exhibits future anxiety for a long time, and they can suffer from or exacerbate the psychological impact of their health conditions. People with high levels of future anxiety tend to think in a pessimism and have impaired problem-solving. There are more likely to experience mental problems, including stress, depression, and anxiety, and reduced well-being [11, 12]. Jannini et al. (2024) found that younger people experiencing high levels of future anxiety were more susceptible to feelings of loneliness, which significantly diminishes their quality of life [13].

In the context of MDs, future anxiety may arise from concerns about reproductive and physical health. Even minor irregular menstruation had been associated with a heightened risk of preeclampsia and negative newborn consequences, even fertility [14]. It could affect their ability to conceive and contribute to future anxiety regarding marriage or childbearing plans [15]. Furthermore, menstrual irregularities are associated with several endocrine disorders, including thyrotoxicosis, hypothyroidism, polycystic ovary syndrome, and diabetes [16], potentially heightening fears about future health. This heightens concerns about long-term health in women with MDs. Conversely, stress and future anxiety can impact the hypothalamic-pituitary-adrenal (HPA) axis, exacerbating menstrual irregularities and creating a feedback circle that further diminishes well-being [17]. There is a complex relationship between MDs and anxiety that simultaneously negatively impacts mental health. Despite the growing recognition of the relationships between MDs and reduced well-being, there are still research gaps in our knowledge of the psychological mechanisms driving this relationship. Prior research has often addressed specific types of MDs, such as dysmenorrhea or irregular menstruation, not for other types of MDs. Additionally, numerous existing studies focus on fluctuating physical or biological factors, such as pain or neurotransmitters and sex hormones, rather than the psychological mechanisms of MDs, which may affect well-being [18–20]. Besides, future anxiety is found to diminish well-being in chronic illnesses like diabetes and cancer [21, 22]. A lack of studies has explicitly tested whether future anxiety mediates the effects of MDs on well-being. Previous studies focused on each type of MD, such as menstrual irregularities or heavy menstrual bleeding.

This research is based on the biopsychosocial model and the transactional model of stress and coping. The biopsychosocial model, proposed by Engel (1977), emphasizes the interplay between biological conditions, psychological responses, and social functioning in shaping health outcomes [23]. In the context of menstrual health, MDs can be viewed as biological stressors that not only cause physical discomfort but may also trigger psychological consequences such as future anxiety, ultimately impairing overall well-being. Similarly, the transactional model of stress and coping (Lazarus & Folkman, 1984) provides a relevant framework: It explains how menstrual disorders are appraised as stressors, and how this appraisal (e.g., future anxiety) leads to negative outcomes (low well-being) [24].

In this study, we investigate the association between MDs and well-being, examining each subgroup separately. This study aims to explore the mediating role of future anxiety in the association between MDs or their subgroups with well-being. We hypothesize that MDs will be associated with lower well-being, and future anxiety will mediate the associations between various MDs and well-being.

## Methods

### Participants and procedure

This cross-sectional study was conducted from June 2021 to May 2022. The data collection method was convenience sampling. This study was part of a larger international project carried out through our research network, which used online surveys on COVID-19-related health knowledge [25]. Portions of the dataset used in the present study have been published previously [26]. Participants were undergraduate and graduate students aged 18 to 48 years from universities across Taiwan. We invited students who were not pregnant at the time of completing the online survey and who had menstruated in the past 6 months. Female students who reported being either currently pregnant, postmenopausal, or having not had a period in the past 6 months and did not provide the consent form were excluded from this study. Information about the study, including online survey questionnaires and consent forms, was sent to students via the university’s email system and posted on social media platforms in Taiwan, including Line and Facebook.

To determine the necessary sample size for the mediation analysis, we consulted the simulation study by Fritz and Mackinnon (2007), which provided recommended sample sizes based on effect sizes of the mediation paths [27]. We used a medium effect size (medium effect size = 0.39) for both paths a (menstrual disturbance → future anxiety) and b (future anxiety → happiness). With a statistical power of 0.8, the minimum sample size was 116 participants. Our final sample consisted of 399 participants, exceeding the minimum and providing adequate power to detect a mediation effect.

### Measurements

#### Assessment of menstrual disorders

Participants reported their menstrual cycles over the last 6 months. Individuals experience MDs if they have at least one of the following symptoms: (1) infrequency (absence of one or more periods/or menstrual cycles exceeding 35 days); (2) irregularity (number of days of the menstrual period lasts or significant variations in menstrual durations); (3) heavy or prolonged bleeding (i.e., bleeding for longer than a week, needing to use double the sanitary protection to control your menstrual flow); (4) abnormal light bleeding (i.e., requiring fewer sanitary products or experiencing shorter bleeding duration than usual); (5) intermenstrual bleeding (spotting between periods); (6) increase in pain/cramps; (7) increase in premenstrual syndrome symptoms (i.e., greater than usual mood swings, feelings of anxiety/depression, tiredness, trouble sleeping, bloating/stomach pain, breast tenderness, changes in appetite or sex drive, or constipation); and (8) one or more missed menstrual periods. The prevalences of types of MDs are presented in Table 1.

**Table 1:** The prevalence of types of menstrual disorders over the last 6 months

	*N*	%
One or more missed menstrual periods		
No	268	67.2
Yes	131	32.8
Infrequent menstruation (i.e., menstrual periods occurring at intervals greater than 35 days)		
No	256	64.2
Yes	143	35.8
Irregular menstruation (i.e., the number of days your menstrual period lasts or the time between each varies significantly)		
No	224	56.1
Yes	175	43.9
Abnormal bleeding or spotting (intermittent bleeding) between normal menstrual periods.		
No	303	75.9
Yes	96	24.1
Heavy or prolonged bleeding (i.e., bleeding for longer than a week, needing to use double the sanitary protection to control your menstrual flow)		
No	364	91.2
Yes	35	8.8
Abnormal light bleeding		
No	330	82.7
Yes	69	17.3
Menstrual pain or cramps		
No	306	76.7
Yes	93	23.3
Premenstrual syndrome symptoms (i.e., greater than usual mood swings, feelings of anxiety/depression, tiredness, trouble sleeping, bloating/stomach pain, breast tenderness, changes in appetite or sex drive, or constipation)		
No	265	66.4
Yes	134	33.6

#### Assessment of well-being

The five-item World Health Organization Well-Being Index (WHO-5) assessed participants’ well-being over the last 2 weeks [28]. This instrument was originally developed and presented at a WHO meeting in Stockholm in 1998 to measure well-being [29]. Individuals self-reported their mood (e.g., feeling cheerful, relaxed, vigorous, or waking up fresh and rested). Each item was recorded from 0 (at no time) to 5 (all of the time). A sum of raw scores ranges from 0 to 25, and a percentage score is formed by multiplying the raw score by 4. Scores of 0 represent the worst possible quality of life, while scores of 100 represent the best. WHO-5 was validated in a Taiwanese population [30]. The Cronbach’s alpha index for this instrument in the study was 0.89.

#### Assessment of future anxiety

The Dark Future Scale, which measures future anxiety, consists of five items [31]. This self-report instrument has a seven-point response scale (0 = decidedly false, 1 = false, 2 = somewhat false, 3 = hard to say, 4 = somewhat false, 5 = true, 6 = decidedly true). The sum of the 5 items computed the scale score; a higher score indicates a higher level of future anxiety. This scale was used in the Taiwanese population [32]. The Cronbach’s alpha coefficient for this instrument in the present study was 0.84.

#### Covariates

Participants provided sociodemographic and health-related information through self-report. Age was assessed with the question, *“How old are you?”*. The education level was recorded as bachelor’s, master’s, or Ph.D. For analysis, education was dichotomized into bachelor’s versus master’s/Ph.D. Participants reported their financial sufficiency, *“How sufficient do you consider the money at your disposal?”* with four response options: *completely sufficient*,* sufficient*,* less sufficient*, and *not sufficient.* Responses were dichotomized as “sufficient” (completely/sufficient) versus “not sufficient” (less/not sufficient). Regarding chronic diseases, individuals who responded “*Do you have a chronic disease or a long-lasting health problem? (This refers to diseases or health problems that last or are expected to last at least 6 months.)”* A “yes” response indicated the presence of a chronic disease. Contraceptive use was assessed with the question, *“Have you recently used any of the following contraceptive methods?”* Response options included oral contraceptive pills, other methods (e.g., intrauterine device, implant), or none. This was recoded as a binary variable (yes vs. no). We also asked participants, “In the last six months, how often have you had difficulties getting to sleep?”. Participants had sleep problems if they answered “yes.’ Additionally, the two-item Patient Health Questionnaire (PHQ-2) was used to assess depression and anhedonia over the past two weeks, with a total score ranging from 0 to 6 [33]. A cut-off score of 3 indicates that participants had depressive symptoms.

All covariates were dichotomized for analysis, including education (bachelor’s vs. master’s/doctoral), financial sufficiency (sufficient vs. not), contraceptive use (yes vs. no), chronic illness (yes vs. no), sleep problems (yes vs. no), and depressive symptoms (yes vs. no).

### Statistical analysis

The SPSS software version 25 was used to analyze the data (IBM Corp, Armonk, NY, USA). Descriptive analysis was performed to determine the mean, standard deviation, frequency, and percentage of the dependent and independent variables. The linear regression analysis investigated the significant factors associated with well-being. To mitigate the effects of confounding factors, variables with a p-value less than 0.20 in the univariable models were adjusted in the multivariable models (*Supplementary Table 1)* [34]. We performed Spearman’s correlation, and for pairs of variables with a rho correlation greater than 0.3, only one variable was retained to avoid multicollinearity (*Supplementary Table 2*). PROCESS 4.3 Model 4 with a 5000 bootstrap sample was utilized to investigate the mediating role of future anxiety on the association between MDs and well-being after adjusting for educational level, birth control, chronic diseases, depression, sufficient money, and sleep problems over the last 6 months. A p-value of 0.05 was applied to all data analyses.

## Results

### The prevalence of menstrual disorders and characteristics of participants

This study found 68.4% of participants experienced at least one menstrual problem, including 35.8% who experienced infrequency, 43.9% who experienced irregularity, 8.8% who suffered heavy or prolonged bleeding, 17.3% who experienced abnormal light bleeding, 24.1% who experienced intermenstrual bleeding, 23.3% who had pain/cramps, 33.6% who had premenstrual symptoms, and 32.8% who missed at least a menstrual period (Table 1). The mean age of the 399 participants was 21.96 ± 5.83 years. Of the study subjects, 80.2% gained a bachelor’s degree, and 71.4% had sufficient financial resources. Regarding health information, there were 8.3% of women used birth control, 13.0% had sleep problems over the past 6 months, 13.3% had chronic medical conditions, and 14.0% reported depressive symptoms. The mean future anxiety score and well-being were 21.26 ± 6.24 and 51.14 ± 19.36, respectively. The mean well-being score was significantly higher among individuals with sufficient financial resources, who had experienced good sleep for over six months, exhibited no depressive symptoms, or had no MDs (*p* < 0.05). Individuals with chronic health conditions or using birth control had lower well-being scores compared to those without, but this difference did not reach statistical significance (*p* > 0.05) (Table 2).

**Table 2:** The characteristics of participants

	*N* = 399	Well-being (mean ± SD = 51.14 ± 19.36)	
	*n* (%)	Mean ± SD	*p*
Age (years) (mean ± SD)			
	21.96 ± 5.83		
Educational level			0.087
Bachelor	320 (80.2)	50.31 ± 19.05	
Master/Ph.D.	79 (17.5)	54.48 ± 20.38	
Sufficient money			< 0.001
No	114 (28.6)	44.70 ± 18.47	
Yes	285 (71.4)	53.71 ± 19.14	
Birth control			0.120
No	366 (91.7)	51.64 ± 19.14	
Yes	33 (8.3)	45.58 ± 20.76	
Sleep problem over 6 months			< 0.001
No	347 (87.0)	52.98 ± 18.64	
Yes	52 (13.0)	38.85 ± 19.78	
Chronic health conditions			0.09
No	346 (86.7)	51.88 ± 18.83	
Yes	53 (13.3)	46.26 ± 22.14	
Depression (PHQ-2 ≥ 3)			< 0.001
No	343 (86.0)	54.02 ± 18.31	
Yes	56 (14.0)	33.50 ± 16.08	
Menstrual disorders			< 0.001
No	126 (31.6)	57.43 ± 18.39	
Yes	273 (68.4)	48.23 ± 19.14	
Future anxiety (mean ± SD)			
	21.26 ± 6.24		

Data was presented with mean ± standard deviation or number (*n*) or percentage (%)

*Abbreviations*: *SD* standard deviation, *Ph.D.* Doctor of Philosophy, *PHQ-2* Patient Health Questionnaire-2

### Correlation between future anxiety, well-being, and menstrual disorders

The results showed significant correlations between various MDs and both future anxiety and well-being. MDs, including missing at least a menstrual period, infrequent, irregular, intermenstrual bleeding, abnormal light bleeding, menstrual pain, and premenstrual symptoms, were positively associated with future anxiety and negatively associated with well-being. Increased premenstrual symptoms had the strongest positive correlation with future anxiety (*r* = 0.26, *p* < 0.01) and a negative correlation with well-being (*r* = −0.29, *p* < 0.01). Additionally, missed menstrual periods show weaker significant relationships with both future anxiety (*r* = 0.11, *p* < 0.05) and well-being (*r* = −0.12, *p* < 0.05). Notably, heavy or prolonged bleeding was not significantly correlated with either future anxiety or well-being. The strong negative correlation between well-being and future anxiety (*r* = −0.43, *p* < 0.01) suggests that greater future anxiety is linked to lower overall well-being (Table 3).

**Table 3:** The correlation between menstrual disorders, future anxiety, and well-being

	Future anxiety	Well-being
Menstrual disorders	0.20**	−0.23**
Infrequent menstruation	0.16**	−0.14**
Irregular menstruation	0.18**	−0.16**
Intermittent bleeding between periods	0.19**	−0.16**
Heavy or prolonged bleeding	0.08	−0.10
Abnormal light bleeding	0.17**	−0.17**
Menstrual pain or cramps	0.17**	−0.14**
Premenstrual symptoms	0.26**	−0.29**
Missed menstrual periods	0.11*	−0.12*
Well-being	−0.43**	

*p-value < 0.05, **p-value < 0.01

### The mediation analysis

**Fig. 1:**
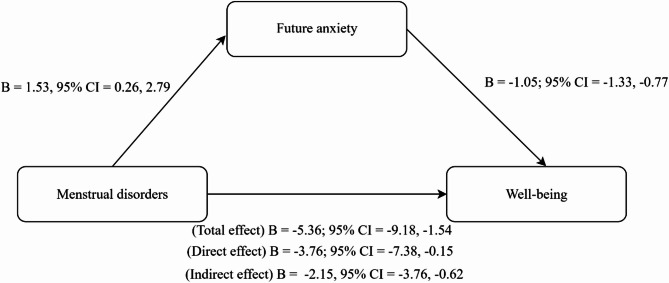
The mediation effect between menstrual disorders, future anxiety, and well-being. B, unstandardized coefficient; CI, confidence interval

Mediation analysis was conducted to examine whether future anxiety mediates the relationship between MDs and well-being after adjusting for educational level, chronic diseases, birth control, depression, financial sufficiency, and sleep problems over the last six months. Results showed that MDs were significantly associated with higher future anxiety (coefficient (B) = 1.53, *p* = 0.018). In turn, higher future anxiety was significantly associated with lower well-being (B = −1.05, *p* < 0.001). The total effect of MDs on well-being was significant (B = −5.36, *p* = 0.006). When future anxiety was included in the model, the direct effects remained significant but weakened (B = −3.76, *p* = 0.041). The significant indirect effect (B = −1.60, 95% confidence interval (95% CI) [−3.21, −0.18]) indicates partial mediation by future anxiety (Fig. 1; Table 4).

**Table 4:** The results of mediation analysis of future anxiety (mediator) on the association between MD or each type of menstrual disorder (independent variables) and well-being (outcome)

	Menstrual disorders	Missed menstrual periods	Infrequency menstruation	Irregularity menstruation	Intermittent bleeding between periods	Abnormal light bleeding	Menstrual pain or cramps	Premenstrual symptoms
	B (95%CI), *p*	B (95%CI), *p*	B (95%CI), *p*	B (95%CI), *p*	B (95%CI), *p*	B (95%CI), *p*	B (95%CI), *p*	B (95%CI), *p*
Total effect	−5.36 [−9.18, −1.54], *p* = 0.006	−1.80 [−5.56, 0.09], *p* = 0.348	−2.11 [−5.86, 1.64], *p* = 0.269	−1.90 [−5.59, 1.79], *p* = 0.313	−3.34 [−7.47, 0.79], *p* = 0.112	−359 (−8.26, 1.09), *p* = 0.132	−5.38 [−9.47, −1.29], *p* = 0.010	−6.61 [−10.40, −2.82], *p* = 0.001
Direct effect	−3.76 [−7.38, −0.15], *p* = 0.041	−1.35 [−4.87, −0.07], *p* = 453	−1.22 [−4.73, 2.30], *p* = 497	−0.92 [−4.38, 2.55], *p* = 0.603	−1.50 [−5.40, 2.41], *p* = 0.451	−2.01 (−6.41, 2.39), *p* = 0.370	−3.25 [−7.13, 0.64], *p* = 0.101	−4.39 [−8.02, −0.77], *p* = 0.018
Indirect effect	−1.60 [−3.21, −0.18]	−0.45 [−1.88, 0.92]	−0.89 [−2.31, 0.42]	−0.98 [−2.40, 0.30]	−1.84 [−3.47, −0.407]	−1.58 (−3.24, −0.06)	−2.14 [−3.81, −0.71]	−2.22 [−3.77, −0.86]

Covariates: educational level, chronic diseases, birth control, depression, sufficient money, and sleep problems over the last 6 months

Table 4 displays the total, direct, and indirect effects of each subgroup and overall MDs on well-being, mediated by future anxiety. Only premenstrual symptoms were negatively associated with lower well-being, both in total effects (B = −6.61, *p* = 0.001) and direct effects (B = −4.39, *p* = 0.018). Future anxiety partially mediates the negative association between premenstrual symptoms and well-being (indirect effects B = −2.22, 95% CI [−3.77, −0.86]). The other MDs had negative associations with well-being, but these effects were not statistically significant overall. After accounting for future anxiety, the direct effects of most MDs on well-being were non-significant, except for premenstrual symptoms. However, the indirect effects on well-being were statistically significant for intermenstrual bleeding between periods (B = −1.84, 95% CI [−3.47, −0.41]), abnormal light bleeding (B = −1.58, 95% CI [−3.24, −0.06), and menstrual pain or cramps (B = −2.14, 95% CI [−3.81 to −0.71]). This indicates that these MDs were negatively associated with well-being primarily through increasing future anxiety rather than through a direct effect. For missed menstrual periods and infrequent or irregular menstruation, mediation by future anxiety was not significant.

## Discussion

The main findings highlight future anxiety as a partial mediator in the relationship between MDs and well-being. Specifically, MDs were significantly associated with higher future anxiety, which, in turn, was linked to lower well-being. Notably, future anxiety partially mediated the association between premenstrual symptoms and well-being. Furthermore, intermenstrual bleeding, abnormally light bleeding, and menstrual cramps or pain did not show direct associations with well-being; however, they were indirectly related to well-being through future anxiety. For missed, infrequent, or irregular menstrual periods, future anxiety did not significantly mediate their link to well-being.

Our findings indicate that MDs were associated with lower well-being, consistent with previous research highlighting the relationship between MDs and mental health challenges. Studies have shown that individuals with MDs are at a heightened risk of experiencing anxiety, depression, and overall psychological distress, which can negatively impact their quality of life [35, 36]. Women with MDs exhibit discomfort, pain, and abnormal hormonal changes, which will increase emotional dysregulation, sensitivity to stress, and decline in well-being [37, 38]. Additionally, menstrual stigma has significant implications for mental and physical well-being, leading to feelings of shame, restrictive practices, misinformation, and reduced quality of life [39]. Menstrual stigma, social norms, and lack of knowledge can reduce help-seeking and social participation, and may lead to emotional responses such as uncertainty, shame, and fear [40, 41]. These feelings can, in turn, worsen concerns about one’s health and future roles (e.g., fertility, womanhood), indirectly contributing to future-oriented issues anxiety. These findings underscore the importance of menstrual health in overall well-being.

The present study highlights the mediating role of future anxiety in the relationship between MDs and well-being. Specifically, future anxiety partially mediated the association between increased premenstrual symptoms and well-being. There is evidence that increased premenstrual symptoms could affect well-being directly, and heightened worries about the future cause some of their negative effects. Premenstrual symptoms are linked to a high risk of exacerbation of depression or anxiety, which negatively impacts well-being [42, 43]. Among women with healthy menstrual cycles, fluctuations in estrogen and progesterone modulate anxiety-related behaviors, contributing to varying levels of anxiety [44].

Moreover, while we did not observe direct associations between intermenstrual bleeding, abnormally light bleeding, and increased menstrual pain or cramps with well-being, these menstrual disorders were indirectly associated with lower well-being through higher levels of future anxiety. This finding suggests that its associations with well-being were mediated by increased future anxiety rather than a direct effect. Individuals with dysmenorrhea may develop anticipatory anxiety due to concerns about symptom unpredictability and long-term reproductive health, underlying health conditions, or disruption of daily activities [9, 45, 46]. Future anxiety, in turn, negatively affects well-being by increasing distress and impairing effective coping mechanisms [47]. Excessive worry about future outcomes has been linked to lower psychological well-being, as it exacerbates stress, anxiety, and emotional distress [48].

Additionally, missed, infrequent, or irregular menstrual periods were not associated with well-being, and future anxiety did not significantly mediate their link to well-being. This finding contrasts with some previous studies, which have found that irregular menstrual cycles are associated with high stress and low well-being [49, 50]. The discrepancy may stem from the difference in study populations. For example, women with polycystic ovary syndrome or who want to have a pregnancy experience more distress than the general population [51]. Moreover, variations in health literacy, access to medical care, and cultural perceptions of menstrual health could influence whether women perceive menstrual irregularities as distressing [52]. Longitudinal research on women with missed, infrequent, or irregular menstrual periods on these contextual factors is needed to help us understand deeply the negative effects on mental health and well-being.

The differences in the relationships between different MDs and well-being may also be linked to underlying physiological and psychological mechanisms. Premenstrual symptoms were closely linked to cyclical hormonal changes, particularly fluctuations in estrogen and progesterone during the luteal phase, which influence neurotransmitter systems such as serotonin and GABA [53, 54]. These neurotransmitters can lead to changes in mood symptoms and anxiety, contributing to lower well-being and increased future-oriented worry. Meanwhile, menstrual irregularities frequently result from conditions such as polycystic ovary syndrome, hypothalamic dysfunction, thyroid disorders, contraceptive use, breastfeeding, excessive physical activity, intrauterine devices, obesity, stress, and smoking [46, 55]. Irregular menstrual cycles are more often associated with chronic hormonal imbalances; physical, mental, social, psychological, and reproductive problems [4]. Abnormal uterine bleeding was caused by diseases related to the structural uterus (polyp, adenomyosis, leiomyoma, malignancy/hyperplasia) or other factors (coagulopathy, ovulatory dysfunction, endometrial, iatrogenic) [56, 57]. This difference in pathological factors may explain the stronger mediating role of future anxiety in the relationship between PMS and well-being, compared to other MDs.

These findings align with prior research, which demonstrates that uncertainty intensifies worry and reduces overall well-being. The results of this study contribute to the understanding of the biopsychosocial model of menstrual health, suggesting that the impact of MDs on health encompasses both biological factors, such as hormonal fluctuations, and psychological factors, including future anxiety. By identifying future anxiety as a key mediating factor, this cross-sectional study offers novel insights into the psychological pathways linking MDs to reduced well-being. Additionally, healthcare providers should screen for future anxiety in patients with MDs to minimize the risk of psychiatric disorders and promote health. The new findings of this study found the role of future anxiety in menstrual health. Besides the strengths, there are some limitations. Menstrual disorders in this study were assessed through self-reported experiences over the past six months, without clinical diagnosis. This may have introduced recall bias or misclassification. Additionally, participants may interpret symptoms differently based on personal norms or cultural perceptions of menstruation. The absence of clinical evaluation also means we could not confirm underlying gynecological conditions (e.g., polycystic ovary syndrome, endometriosis) that may contribute to these symptoms. Future studies should consider incorporating validated clinical criteria and objective assessments where feasible. The cross-sectional design cannot provide conclusive evidence of causality, and future longitudinal studies are necessary to confirm the temporal direction of these associations. The convenience sampling method means that our sample may not be representative or diverse enough to be generalizable to cultural contexts where menstrual stigma varies.

## Conclusions

This study provides valuable insights into the role of future anxiety in mediating the relationship between menstrual disorders and well-being. The findings highlight that while premenstrual symptoms have both direct and indirect effects on well-being, others, like intermenstrual bleeding, abnormal light bleeding, and menstrual pain, primarily influence well-being through increased future anxiety. Additionally, missed, infrequent, or irregular menstrual cycles, as well as heavy or prolonged bleeding, did not show a significant association with well-being. Addressing future anxiety through targeted interventions could help improve overall well-being among individuals experiencing MDs. Further research is needed to explore the mechanisms underlying these relationships and develop effective strategies for mitigating psychological distress associated with menstrual health issues.

## Supplementary Information

Supplementary Material 1.

## Data Availability

The data presented in this study are available on reasonable request from the corresponding author.

## Abbreviations

B: coefficient
CI: Confidence interval
MDs: Menstrual disorders
PHQ-2: Patients Health Questionnaire
SPSS: Statistical package for the social sciences
WHO: World Health Organization
