# Supplementary material for: The association between various menstrual disorders and well-being was mediated by anxiety: a cross-sectional study
Source: BMC Womens Health. 2025 Oct 22;25:508. doi: 10.1186/s12905-025-04053-y (PMC12541953; doi:10.1186/s12905-025-04053-y)
Supplement: Supplementary file 1 — Supplementary Material 1. [file 12905_2025_4053_MOESM1_ESM.docx]

*Supplementary Table 1.* The associated factors of well-being (simple linear regression)

|  | **Well-being** | |
| --- | --- | --- |
|  | B (95% CI) | p |
| Age |  |  |
|  | 0.36 (0.04, 0.69) | 0.029 |
| Educational level |  |  |
| Bachelor | Ref |  |
| Master/Ph.D. | 4.17 (-0.60, 8.94) | 0.087 |
| Sufficient money |  |  |
| No | Ref |  |
| Yes | 9.01 (4.88, 13.14) | < 0.001 |
| Birth control |  |  |
| No | Ref |  |
| Yes | -6.06 (-12.97, 0.84) | 0.085 |
| Sleep problem over 6 months |  |  |
| No | Ref |  |
| Yes | -14.13 (-19.63, -8.64) | < 0.001 |
| Chronic health conditions |  |  |
| No | Ref |  |
| Yes | -5.62 (-11.22, -0.03) | 0.049 |
| Depression (PHQ-2≥3) |  |  |
| No | Ref |  |
| Yes | -20.52 (-25.62, -15.41) | < 0.001 |
| Menstrual disorders |  |  |
| No | Ref |  |
| Yes | -9.19 (-13.20, -5.19) | < 0.001 |
| Future anxiety |  |  |
|  | -1.43 (-1.70, -1.16) | < 0.001 |
| B, unstandardized beta or coefficient; CI, confidence interval; ref, reference; Ph.D., philosophy Doctor; PHQ-2, Patient Health Questionnaire-2. | | |

*Supplementary Table 2.* The correlation between independent variables

|  | Age | Study | Birth control | Sleep | Chronic diseases | Menstrual disorder | Future anxiety | Depression |
| --- | --- | --- | --- | --- | --- | --- | --- | --- |
| Study | **0.61**^**^ |  |  |  |  |  |  |  |
| Birth control | 0.08 | -0.04 |  |  |  |  |  |  |
| Sleep | 0.09 | -0.02 | 0.07 |  |  |  |  |  |
| Chronic disease | 0.13^*^ | 0.05 | 0.10 | 0.22^**^ |  |  |  |  |
| Menstrual disorder | 0.01 | -0.11^*^ | 0.07 | 0.10^*^ | 0.09 |  |  |  |
| Future anxiety | 0.03 | -0.05 | -0.05 | 0.12^*^ | 0.09 | 0.22^**^ |  |  |
| Depression | -0.002 | -0.07 | 0.04 | 0.21^**^ | 0.18^**^ | 0.20^**^ | 0.33^**^ |  |
| Sufficient money | -0.07 | -0.13^**^ | 0.03 | -0.12^*^ | -0.11^*^ | -0.20^**^ | -0.26^**^ | -0.14^**^ |
| *, p-value is less than 0.05; **, p-value is less than 0.01 | | | | | | | | |
